# The Association of Adherence to the Mediterranean Diet with Depression in Older Adults Longitudinally Taking into Account Cognitive Status: Results from the HELIAD Study

**DOI:** 10.3390/nu15020359

**Published:** 2023-01-11

**Authors:** Eirini Mamalaki, Eva Ntanasi, Alexandros Hatzimanolis, Maria Basta, Mary H. Kosmidis, Efthimios Dardiotis, Giorgos M. Hadjigeorgiou, Paraskevi Sakka, Nikolaos Scarmeas, Mary Yannakoulia

**Affiliations:** 1 Department of Nutrition and Dietetics, Harokopio University, 17671 Athens, Greece; 2 1st Department of Neurology, National and Kapodistrian University of Athens Medical School, Eginition Hospital, 11528 Athens, Greece; 3 Department of Psychiatry, National and Kapodistrian University of Athens Medical School, Eginition Hospital, 11528 Athens, Greece; 4 Division of Psychiatry and Behavioral Science, School of Medicine, University of Crete, 70013 Heraklion, Greece; 5 Lab of Cognitive Neuroscience, School of Psychology, Aristotle University of Thessaloniki, 54124 Thessaloniki, Greece; 6 Department of Neurology, Faculty of Medicine, University of Thessaly, 41500 Larissa, Greece; 7 Department of Neurology, Medical School, University of Cyprus, Nicosia 2408, Cyprus; 8 Athens Association of Alzheimer’s Disease and Related Disorders, 11636 Marousi, Greece; 9 Taub Institute for Research in Alzheimer’s Disease and the Aging Brain, The Gertrude H. Sergievsky Center, Department of Neurology, Columbia University, 710 West 168th St, New York, NY 10032, USA

**Keywords:** mediterranean dietary pattern, cognition, dementia, mild cognitive impairment, healthy dietary pattern, elderly

## Abstract

Although research has generally shown a negative association between depression and adherence to the Mediterranean diet (MeDi), the literature related to older adults is controversial, perhaps partially due to the fact that cognitive status has not been considered. The aim of the current work was to investigate the association between MeDi and incident depression in a representative cohort of people, taking into account their cognitive status in multiple ways. The sample was drawn from the HELIAD study, a longitudinal study including a follow-up of 3 years after the baseline assessment. In total, 879 participants without depression at baseline were included (55.4% women, mean age ± Standard Deviation: 73.3 ± 5.0 years). Depression was determined as a score in the Geriatric depression scale ≥6 and/or antidepressant medication and/or clinical diagnosis of depression. Cox proportional hazard models adjusted for age, sex and education were employed. In the basic model, adherence to the MeDi was negatively associated with depression. In the most conservative model, excluding participants with dementia and Mild Cognitive Impairment, and after controlling for the baseline Cognitive Status, each unit (range 0–55) increase in MeDi was associated with a 6.2% decrease in the risk for depression (*p* < 0.001). These findings indicate that MeDi is negatively associated with depression longitudinally in older adults, above and beyond cognitive status.

## 1. Introduction

Depression is a major psychiatric disorder affecting over 300 million people globally, with the estimated number projected to rise [1]. In contrast to its effects on younger adults, depression in older adults is associated with greater adverse effects on physical performance and may affect a person’s ability to live independently [2]; thus, there is special interest in studying depression in adults of older age. 

Modifiable lifestyle factors, such as diet, sleep and physical activity, may constitute promising alternatives to pharmacotherapy to prevent the burden and reduce the incidence and progression of the disease [3]. Among other lifestyle factors, diet has been extensively investigated in relation to depression [4]. Among the most thoroughly investigated dietary patterns is the Mediterranean diet (MeDi); although, studies conducted in older adults have yielded conflicting results. In specific, large-scale, longitudinal investigations have indicated that adherence to the MeDi is negatively associated with depression among older adults [5,6,7]. On the other hand, a systematic review of longitudinal studies, including also younger adults aged 45 years or older, has concluded that there is no relationship between MeDi adherence and depression [8].

These controversial results may at least partially reflect the fact that previous studies of older adults did not account for their cognitive status. Older people suffering from depression usually also have some level of cognitive impairment [9,10] and, vice versa, patients with dementia or Mild Cognitive Impairment (MCI) usually suffer from mood disorders [11,12]. Additionally, as MeDi is also connected with cognitive health [13,14], it becomes clear that cognitive status is a significant factor that may affect the results on the association between MeDi and depression, particularly among older adults.

Other factors related either to the assessment of depression or the dietary assessment may also contribute to the controversial results. The scoring systems used for the evaluation of adherence to the MeDi in some studies were population-specific or were based on non-validated questionnaires [5,15]. Additionally, most of the studies have relied on questionnaires assessing depressive symptoms and have not taken into account the use of anti-depressive medication and/or the clinical diagnosis of depression, which may significantly affect the results. Indeed, it has been found that adherence to the MeDi was protective for depressive symptomatology, only when depressive symptoms were taken into account and not the anti-depressive medications [15]. 

Thus, our aim in undertaking the current study was to account for cognitive health in various and multiple ways in the investigation of the association between MeDi and depression. Thus, we examined the relationship between adherence to the MeDi and specific food groups, characteristic or not of this healthy dietary pattern, with depression incidence in a representative cohort of older individuals, while considering the participants’ cognitive status. 

## 2. Materials and Methods

### 2.1. Samples and Procedures

Participants were community-dwelling older adults aged ≥65 years old, randomly selected from municipality registries for the HELIAD (Hellenic Longitudinal Investigation of Aging and Diet) study, a population-based, multidisciplinary, collaborative study, conducted in an urban area in central Greece (Larissa) and its rural surroundings, as well as in a suburb of Athens. More details about the study procedures and data collection have been reported previously [13,16,17,18]. 

Personal information was gathered from each participant by certified neurologists, neuropsychologists and registered dieticians, involving medical and family history, lifestyle factors and demographic characteristics. Appointments included a thorough neurological examination and neuropsychological assessment, utilizing structured questionnaires, psychometric tests and clinical evaluations. These assessments took place in day centers for older people, the participants’ homes, or municipal public health clinics, after obtaining written informed consent. The study protocol was approved by the Ethics Committees of the University of Thessaly (Approval Code: 138, Approval Date: 8 July 2009) and the National and Kapodistrian University of Athens (Approval Code: 256, Approval Date: 10 May 2021). 

**Figure 1:**
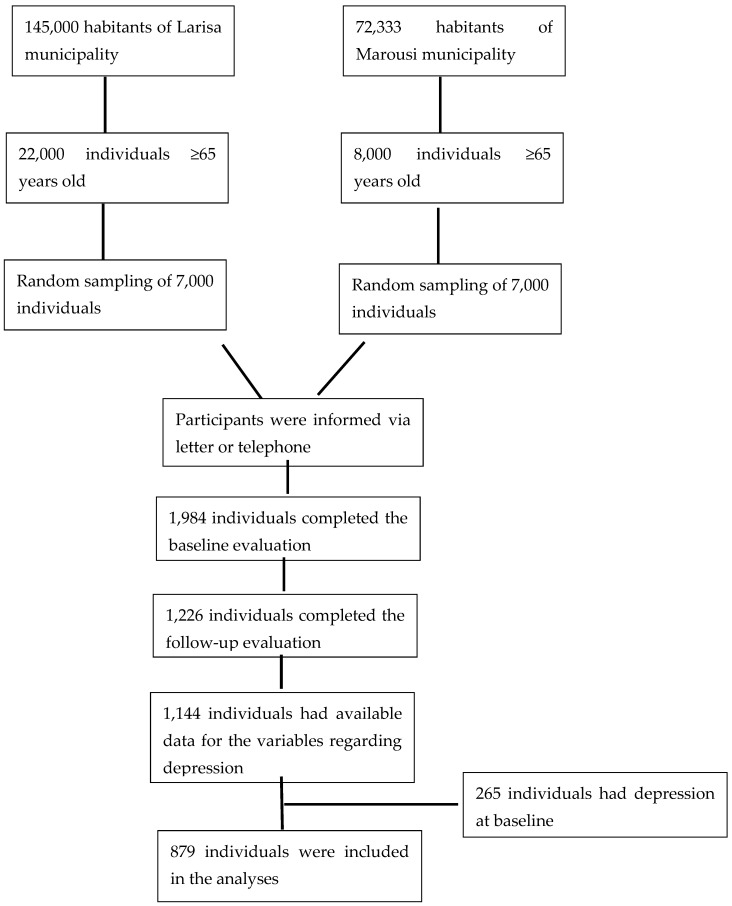
A flowchart of the participants’ sampling.

In the current analysis, 879 participants were included who had a follow-up evaluation, had full data on the assessment regarding depression and did not have depression at baseline. Figure 1 presents the flowchart for this study (Figure 1).

### 2.2. Dietary Assessment

Habitual dietary intake was assessed at baseline using a semi-quantitative Food Frequency Questionnaire, validated for the Greek population [19] and previously used by our group to construct the MeDi score [13,16]. Each participant responded to the questionnaire aided by an investigator and the caregiver, if necessary. Participants indicated the absolute frequency (based on a 6-point scale: “never/rarely”, “1–3 times/month”, “1–2 times/week”, “3–6 times/week”, “1 time/day”, “≥2 times/day”) of consuming particular amounts of food, expressed in units relevant to the type of food (i.e., grams, milliliters, slice, tablespoon, cup). Responses were converted to daily intakes of specific food items as portions/day, i.e., non-refined cereals, cereals, fruits, vegetables, legumes, dairy products, fish, red meat, poultry, alcoholic drinks and sweets.

Adherence to the a priori defined Mediterranean dietary pattern was assessed and yielded the MedDietScore, a composite score based on weekly consumption of 11 food groups, calculated for each participant [20]. These food groups include non-refined cereals, fruits, vegetables, legumes, potatoes, fish, meat and meat products, poultry, full-fat dairy, olive oil use and alcohol. For the food groups whose consumption was characterized as a healthful component of the MeDi, i.e., those with a recommended intake of 3 servings per week or more, such as non-refined cereals, fruits, vegetables, legumes, fish, potatoes and olive oil use, a score of 0 was given when no consumption was reported and scores of 1–5 were given when rare to daily consumptions were reported. In order to accurately reflect the weight of unhealthy food components of the MeDi, a reverse scoring system was used, i.e., assigning a score of 5 for reports of no consumption and extending to 0 for reports of daily consumption. Scoring for alcohol consumption was based on the premise that small amounts of intake were characteristic of the MeDi, but high or no consumption diverged from this dietary pattern. Therefore, a score of 5 was used to indicate intake of less than 300 mL of wine/day but more than 0 mL of wine/day, a score of 0 indicated either no intake or more than 700 mL/day and scores of 1 to 4 reflected intake of 600–700, 500–600, 400–500 and 300–400 mL/day, respectively. All alcoholic beverages were converted into ml of wine, assuming that 12 g of ethanol corresponds to 100 mL of wine. Higher MedDietScores (range: 0–55) indicate a greater adherence to the MeDi. 

### 2.3. Assessment of Variables Related to Depression

Depressive symptoms were assessed with the Geriatric Depression Scale, a 15-item self-report questionnaire regarding depressive symptoms over the past week [21,22]. Additionally, neurologists used information from the clinical examination to determine whether participants met DSM-IV criteria for depression [23] and recorded current medication regimens. The depression variable was a dichotomous one with participants scoring ≥6 in the Geriatric Depression Scale [22], and/or those who had been diagnosed with depression and/or were on antidepressant treatment were considered to suffer from depression. 

### 2.4. Neuropsychological Evaluation and Clinical Diagnosis of Dementia and Mild Cognitive Impairment

At both visits, a neuropsychological evaluation was conducted by trained neuropsychologists with a comprehensive battery of neuropsychological tests, lasting approximately one hour, assessing global cognitive functioning. Specifically, the tests used included the Mini Mental State Examination [24], the Greek Verbal Learning Test (immediate and delayed recall) [25], the Medical College of Georgia Complex Figure Test (copy condition, immediate recall, delayed recall and recognition) [26], a semantic and phonological verbal fluency test [27], subtests of the Greek version of the Boston Diagnostic Aphasia Examination short form, namely the Boston Naming Test short form, and selected items from the Complex Ideational Material Subtest [28], the Trail Making Test Part [29], an abbreviated form of Benton’s Judgment of Line Orientation [30], the Clock Drawing Test [31], anomalous sentence repetition [28], a graphical sequence task and motor programming [26].

Subsequently, we calculated z-scores for the raw data gleaned from each neuropsychological test variable, based only on those participants without a dementia or a mild cognitive impairment (MCI) diagnosis. We then summed up these z-scores to calculate a composite score reflecting overall abilities (Global Cognition score). Diagnoses of dementia and MCI (in accordance with international criteria [23,32,33]) were determined in consensus meetings of all study investigators, based on the data gleaned from both the neuropsychological assessment and the structured neurological examination.

### 2.5. Other Variables

Participants at baseline were asked if they smoke (0; no, 1; yes) and how many people they live with; a dichotomous variable was created indicating whether the participant lived alone or with others (0: alone, 1: with others). Additionally, neurologists recorded all the chronic conditions the participants had, and we computed a variable indicating the sum of these conditions at baseline. Height and weight were measured at baseline on a leveled platform scale and a wall-mounted stadiometer, to the nearest 0.5 cm and 0.5 kg, respectively. Body Mass Index (BMI, kg/m^2^) was calculated by dividing the weight in kilograms by the height in meters squared.

Additionally, at baseline, we evaluated physical activity using the validated Athens Physical Activity Questionnaire (APAQ) [34]. Participants were asked about their participation in various activities in the last week, as well as time spent on occupational, household and recreational activities, sedentary activities and sleep time. Based on the specific metabolic equivalent (MET), which corresponds to each of these activities, energy expenditure was calculated based on the participant’s body weight in kilograms divided by 60. The physical activity variable was expressed as total MET-min/day, excluding sleep.

### 2.6. Statistical Analyses

Statistical analyses were performed using SPSS 26 (SPSS, Chicago, IL, USA). Participants with depression at baseline were excluded (N = 265) from all analyses. The significance level (alpha) was set at *p* ≤ 0.05. The characteristics of the participants were expressed as mean ± standard deviation or as percentages; comparisons were conducted using analysis of variance for continuous variables and Pearson’s x^2^ for categorical variables.

We calculated Cox proportional hazards models with depression incidence as the dichotomous outcome, and adherence to the MeDi as the independent variable. The time-to-event variable was the number of years from the baseline evaluation to the visit where depression was determined; participants who did not develop depression were censored at the time of their last follow-up visit. Except for the basic model, which was adjusted for age, sex and years of education, we constructed more models, in order to thoroughly account for cognitive status: (i) model 1: basic model but further excluding participants with dementia at baseline (N = 28), (ii) model 2: model 1 but further excluding participants with MCI at baseline (N = 118), (iii) model 3: model 2 but further adjusting for baseline Global Cognition Score and, subsequently, a survival plot for depression incidence was made. In all models, MedDietScore was entered into the models both as a continuous and a categorical variable, as tertiles (comparing the first versus other tertiles). We treated age, years of education, baseline Global Cognition Score and the follow-up interval duration as continuous variables, whereas sex (female vs. male as the reference) was treated as a categorical variable.

In the subsequent analyses, we used model 3 to investigate the association of the individual food groups (independent variables) in a continuous form in portions/day entered in the models individually, with depression incidence as the outcome. Additionally, in a supplementary analysis, we employed Cox proportional models, again using model 3, with the individual components of depression variables and adherence to the MeDi, as the independent variable, in a continuous form. 

Finally, in a supplementary analysis, we used the basic model with depression incidence as the outcome and adherence to the MeDi as the independent variable, but we additionally adjusted it for BMI, if the participant lived alone, smoking status, sum of co-morbidities and physical activity. BMI, physical activity and the sum of co-morbidities were used as continuous variables, whereas the smoking status and if the participant lived alone variables were used as categorical variables.

## 3. Results

In the current analysis, 879 participants were included, with a mean follow-up interval of 3.0 ± 0.8 years. Of those, 170 individuals developed depression at follow-up (19.3% of the participants). A greater proportion of the participants who developed depression also developed MCI and dementia at follow-up, and were women, compared with those who did not develop depression. Additionally, they had lower values of MedDietScore, indicating lower adherence to the MeDi, and they consumed fewer portions of fruits, vegetables and alcoholic drinks per day. Demographic characteristics for all participants, as well as for participant groups based on depression, are presented in Table 1. Of the participants having depression at follow up, 23.2% received anti-depressants, 64.1% had a score in the Geriatric Depression Score ≥ 6, and 57.0% had a clinical diagnosis of depression at follow-up; a total of 58.5% had one of the three components of the depression, 37.3 had two components and 3.5 had all of them (results not shown).

**Figure 2:**
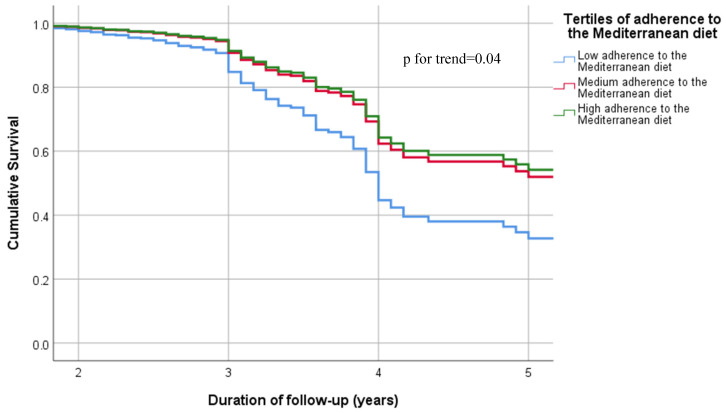
Cumulative Survival Curves from Cox analysis for depression incidence according to the tertiles of adherence to the Mediterranean diet. The models are adjusted for age, sex, years of education and baseline Global Cognition Score.

Results from Cox models in the Basic model (participants without depression at baseline and adjusting for age, sex and years of education) between adherence to the MeDi and depression incidence demonstrated a negative association between adherence to the MeDi and depression. After excluding participants with dementia and MCI and further adjusting for baseline Global Cognition Score the results did not change. In specific, each unit increase in the MedDietScore, indicating greater MeDi adherence, was associated with an 6.2% decrease in the risk for depression (*p* = 0.001). When MeDi adherence was entered into the models as tertiles, those in the highest tertile, that is, those having the greatest MeDi adherence, had 46% lower risk for depression for every unit increase in the MedDietScore, compared with those having the lowest MeDi adherence (*p* = 0.006), with a significant trend for dose–response (Table 2, Figure 2).

In Supplementary Table S1, we repeated the analysis for the Basic Model, but we additionally accounted for smoking status, if the participant lived alone, body mass index, physical activity and number of co-morbidities, and the results remained the same (Supplementary Table S1).

Regarding the consumption of individual food groups in the models adjusted for age, sex, years of education and baseline Global Cognition Score, the consumption of fruits and alcoholic drinks was negatively associated with depression incidence; for every portion increase in fruits and alcoholic drinks, the risk for depression decreased by 15.2% and 31.3%, respectively (*p* < 0.05 for both) (Table 3).

In Supplementary Table S2, the results from Cox proportional models between the individual components of depression and adherence to the MeDi are presented in participants without dementia and MCI at baseline. The results showed that adherence to the MeDi is negatively associated with all the components, i.e., use of anti-depressive medication, score ≥6 in the Geriatric Depression Scale and clinical diagnosis of depression, after adjusting for age, sex, years of education and baseline Global Cognition Score (Supplementary Table S2).

## 4. Discussion

The aim of the study was to examine the association between MeDi adherence and depression incidence, after extensively controlling for cognitive status using multiple methods. We found that, after applying a very conservative approach of excluding participants having dementia and MCI and adding to the models the baseline Global Cognition Score, adherence to the MeDi was negatively associated with depression incidence; thus, older adults having greater adherence to the MeDi also have a lower risk for depression, above and beyond cognitive status. Regarding the individual food groups characterizing, or not, the MeDi, negative associations were found for fruits and alcoholic beverages. 

The prevalence of depression is greater in older people compared with younger individuals [35]. A recent meta-analysis found that the prevalence of depression in older adults is around 28% [36]. In any case, it is well known that individuals with depression have compromised cognitive health [37,38] and that the prevalence of depression in older people having cognitive impairment is higher compared with those having normal cognitive functions [11,12]; however, it is not known whether depression is a risk factor for compromised cognitive health or whether depression constitutes a part of the long preclinical phase of the disease. Additionally, keeping in mind that adherence to the MeDi is positively connected with cognitive health [13,14], it is of great importance cognitive function to be accounted for when exploring the connection of diet with depression. We adopted a thorough methodology to account for this significant parameter; thus we are pretty confident that the association found was for depression per se, and not confounded by the cognitive status of the participants. 

The underlying mechanisms connecting MeDi and depression are not fully established. A possible explanation is related to the modulation of the inflammatory level [39], bearing in mind that the MeDi reduces body inflammation [40]. Other hypotheses include pathways involved in the reduction of oxidative stress and the modulation of microbiota–gut–brain axis pathways [39]. Additionally, specific micro-nutrients abundant in the MeDi, such as vitamins C and E, as well as poly-unsaturated fatty acids, may be involved in the mechanisms [41]. In any case, it should be noted that the action that the adherence to the MeDi exerts is most likely not restricted to only one pathway, but it is multifaceted. This assumption is enhanced by the fact that adherence to the MeDi was negatively connected with all the components used for the computation of the depression variable in our study, i.e., depressive symptoms, anti-depressive medication and the clinical diagnosis of depression set by neurologists after the clinical examination. Thus, this may further suggest that the MeDi exerts its actions by different pathways affecting different aspects of mental health.

Regarding the individual food groups, negative associations were found for fruits and alcoholic drinks with depression incidence. Although most studies on alcohol intake have explored disorders involving alcohol use, rather than lower intake levels, studies in younger adults have indicated that the association between alcohol consumption and depression is U-shaped [42]; whereas, results from other studies have shown that moderate alcohol consumption was more prevalent among non-depressed older people and was associated with reduced depression development [38,43]. It should be noted that in our study, the mean portion of alcoholic drinks was low, and the maximum consumption was within the acceptable range, so is safe to assume that there was no alcohol overconsumption that could negatively affect mood and result in mood disorders. The mechanisms implicated may be the reduction of inflammation and the neuroprotection that some non-alcoholic components of alcoholic drinks may exert [44].

When it comes to fruits, other studies have found similar results; increased fruit consumption is associated with reduced depression incidence [8,45]. The exact mechanisms that account for these relationships are not well investigated, but there is evidence that anti-oxidants that are abundant in fruits, such as vitamins C, E and folate may explain, in part, the observed relationships [46]. Additionally, other nutrients included in fruits act as co-factors for neurotransmitters, significant in mood regulation [47]. It has been also suggested that in older adults inflammation markers are connected with low fruit consumption [48]; thus, the association of fruits with depression may be explained through neuroinflammation.

When interpreting the findings of the present study, one should bear in mind both its strengths and its limitations. Starting with the limitations, the follow-up interval was relatively short, making it difficult to extract conclusions on cause and causality. What is more, the assessment of diet and depression was based on self-reporting; thus, factors such as social desirability or memory lapses related to aging may have influenced responding, leading to an increased risk for report. However, we tried to elucidate this parameter by excluding from the sample participants with dementia and/or MCI and further controlling for their cognitive status. Additionally, for assessing depression, we used different components, and the diagnosis was not set by psychiatrists. However, the diagnosis was determined based on international criteria (DSM-IV) and we explored whether this influences the results by running different models with each of these components. 

At the same time, the study has important strengths. Unlike previous investigations, clinical assessment by dementia experts in the present study enabled a fine-tuned classification of the participants’ cognitive status. Additionally, we excluded from the sample participants with depression at baseline, which strengthens the confidence in our results. Finally, dietary data were collected using instruments validated in the population under investigation, and the score used for calculating adherence to the MeDi, the MedDietScore, is based on an a priori threshold, regardless of the consumption of the population under investigation [20].

## 5. Conclusions

In summary, the results of the present study indicate that MeDi adherence is negatively associated with depression incidence in older individuals, independent of their cognitive status. More studies are needed to clarify the mechanisms behind this relationship and should take into account other characteristics of the investigated populations, such as genetic predisposition to depression. However, this finding may be useful for designing and implementing population-based prevention programs. Additionally, the findings may be used in clinical practice towards improving the symptoms and, even preventing, mood disorders in older adults. 

## Figures and Tables

**Table 1:** Baseline demographic, dietary characteristics and adherence to the Mediterranean diet of all participants and based on the diagnosis of follow-up.

	All Participants N = 879	Participants without Depression at Follow Up N = 709	Participants with Depression at Follow Up N = 170	*p*-Value *
Age (years)	73.3 ± 5.0	73.1 ± 4.9	74.0 ± 5.2	0.057
Sex (% women)	55.4	54.5	68.3	**0.003**
Education (years)	8.3 ± 5.0	8.5 ± 4.9	7.2 ± 4.9	**0.002**
Duration of follow-up (years)	3.0 ± 0.8	3.0 ± 0.8	3.1 ± 0.8	0.211
Diagnosis of MCI at follow-up (%yes)	17.3	14.3	21.4	**0.033**
Diagnosis of dementia at follow-up (%yes)	4.9	3.9	9.0	**<0.001**
Global Cognition Score	−0.1 ± 0.8	−0.1 ± 0.7	−0.3 ± 0.8	**0.006**
Living alone (%yes)	16.2	16.3	19.5	0.321
Smoking status (%yes)	10.9	10.9	11.2	0.123
Body Mass Index (kg/m^2^)	28.8 ± 4.3	28.8 ± 4.2	28.7 ± 4.5	0.777
Number of co-morbidities	2.0 ± 1.4	2.0 ± 1.4	2.2 ± 1.4	0.221
Physical Activity (/200 MET-min/day)	65.6 ± 9.1	65.5 ± 9.1	66.0 ± 9.1	0.521
MedDietScore (0–55)	33.7 ± 4.5	34.1 ± 5.0	32.3 ± 5.0	**<0.001**
Non refined cereals (portions/day)	1.0 ± 1.4	1.0 ± 1.5	0.9 ± 1.5	0.605
Cereals (portions/day)	4.7 ± 2.0	4.6 ± 2.0	4.9 ± 1.8	0.149
Fruits (portions/day)	2.1 ± 1.3	2.1 ± 1.3	1.8 ± 1.2	**0.003**
Vegetables (portions/day)	2.0 ± 0.9	2.0 ± 0.9	1.8 ± 0.8	**0.002**
Legumes (portions/day)	0.5 ± 0.3	0.5 ± 0.3	0.4 ± 0.3	0.456
Dairy products (portions/day)	1.7 ± 0.9	1.7 ± 0.9	1.6 ± 0.8	0.081
Fish (portions/day)	0.6 ± 0.4	0.6 ± 0.4	0.6 ± 0.4	0.522
Red meat (portions/day)	0.8 ± 0.5	0.8 ± 0.5	0.8 ± 0.5	0.642
Alcoholic drinks (portions/day)	0.4 ± 0.7	0.5 ± 0.8	0.2 ± 0.5	**<0.001**
Sweets (portions/day)	0.4 ± 0.5	0.4 ± 0.5	0.2 ± 0.4	0.651

* refers to the comparison between participants with depression and those without depression. Continuous variables are presented as mean values ± standard deviation and categorical variables as relative (%) frequencies. MCI: Mild Cognitive Impairment, MedDietScore: Score indicating adherence to the Mediterranean diet. Bold letters indicate statistical significance (*p* < 0.05).

**Table 2:** Results from Cox models calculating the association between adherence to the Mediterranean Diet both as continuous variable and as tertiles (independent variables), with depression incidence (dependent variable) in participants without depression at baseline.

	Adherence to the Mediterranean Diet as a Continuous Variable		Adherence to the Mediterranean Diet as Tertiles			
	HR (95% CI)	*p*		HR (95% CI)	*p*	*p* for Trend
Basic Model (N = 879)	0.947 (0.915–0.981)	**0.002**	1st (reference)			**0.002**
			2nd	0.577 (0.393–0.878)	**0.009**	
			3rd	0.535 (0.350–0.816)	**0.004**	
Model 1 (N = 854)	0.948 (0.915–0.98)	**0.003**	1st (reference)			**0.002**
			2nd	0.588 (0.393–0.878)	**0.009**	
			3rd	0.537 (0.350–0.819)	**0.004**	
Model 2 (N = 736)	0.937 (0.903–0.973)	**0.001**	1st (reference)			**0.003**
			2nd	0.590 (0.395–0.885)	**0.009**	
			3rd	0.539 (0.359–0.816)	**0.004**	
Model 3 (N = 736)	0.938 (0.903–0.974)	**0.001**	1st (reference)			**0.004**
			2nd	0.586 (0.391–0.879)	**0.010**	
			3rd	0.549(0.359–0.840)	**0.006**	

Basic model: adjusted for age, sex and years of education. Model 1: Basic model excluding participants with dementia at baseline. Model 2: Basic model excluding participants with dementia and Mild Cognitive Impairment at baseline. Model 3: Μοdel 2 further controlling for Baseline Global Cognition Score. Bold letters indicate statistical significance (*p* < 0.05). HR: Hazard Ratio, CI: Confidence Interval.

**Table 3:** Results from Cox models evaluating the association between consumption of food groups (independent variables) and depression incidence (dependent variable) in participants without depression, and with normal cognitive function at baseline.

Consumption of Specific Food Groups	HR (95% CI)	*p*
Non refined cereals	0.907 (0.799–1.029)	0.133
Fruits	0.848 (0.741–0.972)	**0.018**
Vegetables	0.847 (0.697–1.029)	0.094
Legumes	0.972 (0.480–1.966)	0.937
Fish	0.774 (0.492–1.217)	0.267
Red meat	0.795 (0.548–1.152)	0.225
Alcoholic drinks	0.687 (0.472–0.991)	**0.047**

The models are adjusted for age, sex, years of education and baseline Global Cognition Score. Bold letters indicate statistical significance (*p* < 0.05). HR: Hazard Ratio, CI: Confidence Interval.

## Data Availability

The data presented in this study are available on reasonable request from the corresponding author.

## Supplementary Materials

The following supporting information can be downloaded at: https://www.mdpi.com/article/10.3390/nu15020359/s1, Table S1: Results from Cox models that evaluated the association between adherence to the Mediterranean Diet both as continuous variable and as tertiles (independent variables) with depression incidence (dependent variable) in participants without depression at baseline; Table S2: Results from Cox models that evaluated the association between adherence to the Mediterranean diet (independent variables) with the individual components of depression variable (dependent variables) in participants without depression, and with normal cognitive function at baseline.
