# Supplementary material for: The Association of Adherence to the Mediterranean Diet with Depression in Older Adults Longitudinally Taking into Account Cognitive Status: Results from the HELIAD Study
Source: Nutrients. 2023 Jan 11;15(2):359. doi: 10.3390/nu15020359 (PMC9865507; doi:10.3390/nu15020359)
Supplement: Supplementary file 1 [file nutrients-15-00359-s001.zip › nutrients-2106236-supplementary.pdf]

Supplementary Table S1. Results from Cox models that evaluated the association between adherence to the Mediterranean Diet both as continuous variable and as tertiles (independent variables) with depression incidence (dependent variable) in participants without depression at baseline.

Adherence to the Mediterranean Diet as a Continuous Variable		Adherence to the Mediterranean Diet as Tertiles			
**HR (95% CI)**	**p**		**HR (95% CI)**	p	p for trend
0.949 (0.918-0.986)	**0.004**	1st (reference)			**0.005**
		2nd	0.554 (0.358-0.857)	0.008	
		3rd	0.552 (0.361-0.845)	0.003	

The models were adjusted for age, sex, years of education, if the participant lived alove, number of co-morbidities, smoking status, physical activity and Body Mass Index; Bold letters indicate statistical significance ($p < 0.05$). HR: Hazard Ratio, CI: Confidence Interval.

Supplementary Table S2. Results from Cox models that evaluated the association between adherence to the Mediterranean diet (independent variables) with the individual components of depression variable (dependent variables) in participants without depression, and with normal cognitive function at baseline.

**Consumption of Specific Food Groups**	**HR (95% CI)**	**p**
Use of anti-depressant medication (N = 705)	0.877 (0.801-0.961)	**0.005**
Score of Geriatric Depression Scale ≥6 (N = 750)	0.933 (0.885-0.983)	**0.010**
Clinical Depression (N = 744)	0.901 (0.848-0.958)	**0.001**

The models are adjusted for age, sex, years of education and Baseline Global Cognition Score Bold letters indicate statistical significance (p<0.05). HR: Hazard Ratio, CI: Confidence Interval.
